# Antibiotic susceptibility of mastitis pathogens in Finnish dairy cows in 2022–2023: concerning changes in the penicillin susceptibility of *Streptococcus uberis*

**DOI:** 10.1186/s13028-026-00875-4

**Published:** 2026-08-21

**Authors:** Suvi Nykäsenoja, Thomas Grönthal, Päivi Rajala-Schultz, Tiina Autio

**Affiliations:** 1 Microbiology Unit, Laboratory and Research Division, Finnish Food Authority, Mustialankatu 3, FI-00790 Helsinki, Finland; 2 Animal Health Diagnostic Unit, Laboratory and Research Division, Finnish Food Authority, Mustialankatu 3, FI-00790 Helsinki, Finland; 3 Present Address: Faculty of Veterinary Medicine, University Teaching Hospital, University of Helsinki, Helsinki, Finland; 4 Department of Production Animal Medicine, Faculty of Veterinary Medicine, University of Helsinki, Paroninkuja 20, FI-04920 Saarentaus, Finland; 5 Animal Health Diagnostic Unit, Laboratory and Research Division, Finnish Food Authority, Neulaniementie 4, FI-70210 Kuopio, Finland

**Keywords:** Antibiotic resistance, Antibiotic susceptibility, Mastitis, *Streptococcus uberis*

## Abstract

**Background:**

Intramammary infections are the most common reason for antibiotic use in dairy cattle. A multiplex PCR has become the mainstay in determining the causative mastitis pathogens in Finland. However, this method does not allow the monitoring of antibiotic resistance among mastitis pathogens. The aim of this study was to assess antibiotic susceptibility among common bacterial pathogens isolated from mastitis milk specimens in Finland. Specimens were collected on dairy farms from cows suffering from mastitis and analysed with the PathoProof™ Mastitis Complete-16 PCR assay. Specimens with moderate or large amounts of *Staphylococcus aureus*, *Streptococcus agalactiae*, *Streptococcus uberis*, *Streptococcus dysgalactiae*, *Escherichia coli*, *Klebsiella* sp. and non-aureus staphylococci (NAS) were cultured and susceptibility testing was performed with the broth microdilution method. Susceptibility results were categorised as wild type (WT) and non-wild type (NWT) according to epidemiological cut-off values. Resistance to penicillin among *S. aureus* and NAS was assessed based on phenotypic beta-lactamase production. Only one bacterial isolate was included per species and farm.

**Results:**

In total, 502 bacterial isolates from 220 farms were tested for susceptibility. The most common bacterial species isolated was *S. aureus* (*n* = 126), followed by NAS (*n* = 124), *S. uberis* (*n* = 79), *S. dysgalactiae* (*n* = 74) and *E. coli* (*n* = 70). The proportions of NWT isolates were generally low, with the notable outlier being *S. uberis*, for which 42% (95% CI: 31.5–52.8) of the isolates was classified as NWT to penicillin. Furthermore, 38% (95% CI: 29.9–46.7) of the NAS isolates produced beta-lactamase. Only 4% (95% CI: 1.7-9.0) of the *S. aureus* isolates produced beta-lactamase, and no methicillin resistance was detected. All *E. coli* were classified as WT to fluoroquinolones, and no extended-spectrum beta-lactamase (ESBL)-producing isolates were discovered.

**Conclusions:**

Antibiotic susceptibility among bacteria in clinical mastitis is favourable. However, the proportion of *Streptococcus uberis* isolates classified as NWT to penicillin has increased over the past ten years, and continued surveillance is warranted.

**Supplementary Information:**

The online version contains supplementary material available at https://doi.org/10.1186/s13028-026-00875-4.

## Background

Intramammary infections (IMI) are the most common bacterial infections of dairy cattle and have long been the most common reason for antibiotic use in dairy cows [1]. With increasing concerns over the impacts of antibiotic use, it is essential to reduce antibiotic consumption, as well as to monitor antimicrobial resistance (AMR) and the underlying molecular resistance mechanisms in pathogens. Furthermore, mastitis and antibiotic use are a financial burden to farmers due to both treatment costs and lost revenue from discarded milk.

The most commonly encountered bacteria causing IMI in Nordic countries include Gram-positive cocci, such as *Staphylococcus aureus*, non-aureus staphylococci (NAS, e.g., *S. simulans* and *S. chromogenes*), *Streptococcus uberis* and *Streptococcus dysgalactiae*. *Escherichia coli* is the most common Gram-negative bacterium causing IMI [2–7]. AMR among mastitis pathogens differs greatly between countries. The Nordic countries have traditionally been a region of fairly low AMR due to a number of preventive measures, such as strict regulations on the sales and use of antibiotics for mastitis, national guidelines focusing on the use of narrow-spectrum antibiotics, early diagnosis and adaptation of therapy, the low use of antibiotics in dry-cow therapy (DCT), as well as a coordinated effort by farmers, veterinarians and industry promoting udder health and reducing the use of antibiotics [8]. As an example, in recent Nordic studies, penicillin resistance among *S. aureus* isolates obtained from mastitis cases has ranged between 2.6% [4] and 17.5% [3]. According to a recent meta-analysis [9], the global pooled estimate of the prevalence of penicillin G resistance among *S. aureus* isolated from bovine milk was 45% (95% CI: 14.5–48.7). Resistance to other antibiotics in the Nordic countries is commonly even lower than that described for penicillin and *S. aureus* [3–6].

Polymerase chain reaction (PCR) assays that target the most common mastitis pathogens are currently very commonly in use in Finland and bacterial culture is rarely performed [8]. While this enables farmers and veterinarians to receive results faster, the PCR assays do not provide information on the susceptibility of the pathogens, apart from screening for the *blaZ* gene, which induces penicillin resistance in *Staphylococcus* spp. [8]. Therefore, very little data are available on AMR in pathogens causing IMI in Finland. The goal of this prospective study was to determine the proportion of non-wild type (NWT) isolates, based on epidemiological cut-off values, among the most common mastitis pathogens in Finnish dairy cattle. In addition, penicillin resistance in staphylococci was assessed based on phenotypic beta-lactamase production.

## Methods

### The dairy industry and milk quality in Finland

In 2022, there were 4,750 dairy farms in Finland, with 246,500 dairy cows [10]. The average farm size was 52 cows [11], while 555 farms had one hundred cows or more. Milk from Finnish cows is graded based on its quality, where a somatic cell count (SCC) of <250,000/mL and a bacterial count of <50,000/mL are considered the highest quality. In 2022, over 97% of Finnish milk produced was of the highest quality, and the national average bulk tank somatic cell count was 133,000 cells/mL [12]. In Finland, most dairy cows (70%) are kept in free-stall barns and over half of the milking is performed by automatic milking systems [10].

### Study farms and selection of samples

The target farms, totalling approximately 4,000 farms, were milk producers of the major Finnish dairy company Valio Ltd. This company is a farmer-owned cooperative with several regional processing co-ops and members across the entire country, and it processes approximately 80% of all the milk produced in Finland. The study farms (*n* = 600, with an expected participation of 300 farms) were selected based on the following criteria: (1) farms with over 50 cows were included and (2) the sampling was stratified per regional dairy cooperative and weighted by the number of farms in the region (i.e., more farms were selected in larger cooperatives). The selection of farms was randomized using the randomization tool in Microsoft Excel. In addition, farms with more than five *Klebsiella* spp. or five *S. agalactiae* findings, as well as all new *S. agalactiae*-positive farms in 2021 were selected.

The sample size per bacterial species was calculated using the Epitools sample size calculator [13] based on previous Finnish studies, with assumptions that 20% of *S. aureus*, 30% of NAS and 5% of streptococcus isolates would be categorised as non-wild type (NWT) to penicillin, and 5% of *E. coli* would be categorised as NWT to ampicillin, with a desired precision of 5%. Test sensitivity and specificity were not accounted for, as specimens were selected using PCR results. The goal was to obtain the following number of strains per bacterial species or group: NAS (*S. epidermidis*, *S. simulans*, *S. chromogenes*, *S. haemolyticus*): 323 in total; *Staphylococcus aureus*: 246; *Streptococcus agalactiae*: all detected; *Streptococcus uberis*: 73; *Streptococcus dysgalactiae*: 73; *Escherichia coli*: 73; *Klebsiella oxytoca/pneumoniae*: all detected. Specimens in which a bacterium of interest was detected by PCR in at least a moderate amount (++ or +++ according to PathoProof™ software) and which were positive for at most two bacterial species were selected for culture. For each farm, the first isolate per bacterial species was included in the study.

### Sampling

Study farms received packages containing sampling tubes as well as a consent form, sampling instructions and information about the project and privacy policy. Specimens from both clinical and subclinical mastitis were accepted. However, milk samples collected at dry-off and as controls after antibiotic therapy were not included in the study. The sampling was carried out between December 2021 and July 2023. Personnel in the selected study farms took duplicate milk samples using aseptic technique from quarters with clinical signs and/or elevated SCC, one with bronopol preservative (for PCR) and one without (for culture and subsequent susceptibility testing). Farms were instructed to take only one quarter milk sample per cow. When two or more quarters were infected, the sample was to be taken from the quarter with the most severe signs of inflammation. Samples were cooled and sent via milk collection trucks to the Valio Regional Laboratory in Seinäjoki, and the samples arrived at the laboratory refrigerated within 48 h of sampling.

### PCR for mastitis pathogens and bacteriological examination

Multiplex real time PCR for the 15 most common mastitis pathogens was performed using the PathoProof™ Complete-16 Kit (Thermo Fisher Scientific, Vantaa, Finland) according to the manufacturer’s instructions in the Valio Regional Laboratory in Seinäjoki. Upon receipt in the laboratory, milk specimens for bacterial culture were frozen (-20 °C) pending PCR results. Frozen milk samples were shipped on ice from Valio overnight to the Finnish Food Authority in Kuopio. Samples with at least a moderate amount of bacterial species DNA of interest in PCR were thawed and cultured on Columbia agar (Oxoid Ltd., Basingstoke, UK) supplemented with 5% sheep blood. Agar plates were incubated in a normal atmosphere at + 35 ± 1 °C for 48 h. If one to two bacterial species were detected, a minimum of five colony forming units (CFU) were needed to warrant further examination. For *S. agalactiae*, only one CFU sufficed. The sample was rejected as mixed growth if it grew three or more species. Isolates of interest were pure cultured and identified using matrix-assisted laser desorption ionization time-of-flight mass spectrometry (MALDI-TOF) on a Bruker microflex LT device (Bruker Daltonics, Bremen, Germany) following the direct smear method. Identification was performed according to the manufacturer’s instructions using a commercially available MALDI Biotyper (MBT) library for identification of bacteria and fungi (MBT Compass Library Revision H). Isolates of interest were stored at -80 °C for further testing. A score of > 2.00 was required for species identification. Staphylococci that were not reliably identified (score < 2.00) with MALDI-TOF were further tested using API ID 32 Staph (bioMérieux, Marcy-l’Etoile, France) according to the manufacturer’s instructions. In addition to MALDI-TOF, *Staphylococcus borealis* isolates were identified via calculation of the average nucleotide identity (ANI) by whole genome sequencing (WGS, see below).

### Susceptibility testing

The susceptibility testing of bacteria was performed with the broth microdilution method according to the Clinical and Laboratory Standards Institute (CLSI) standard VET01 5th edition [14] using custom-made Sensititre™ microtiter plates (Thermo Fisher Scientific, East Grinstead, UK). The tested antibiotics per bacterial species are presented in Additional file 1. Susceptibility results (minimum inhibitory concentrations, MICs) were interpretated as wild type (WT) and non-wild type (NWT) using (tentative) epidemiological cut-off values ((t)ECOFFs, available on 14.2.2025) defined by the European Committee on Antimicrobial Susceptibility Testing (EUCAST). *Escherichia coli* ATCC 25922, *Staphylococcus aureus* ATCC 29213 and *Streptococcus pneumoniae* ATCC 49619 were included as quality controls for susceptibility testing on at least a weekly basis. The Epitools calculator (Wilson method) was used for determining the confidence intervals for a proportion where relevant [13].

### Determination of beta-lactamase production and detection of *blaZ* and *mecA*/*mecC* genes

Beta-lactamase production of staphylococci isolates was analysed with Cefinase™ disks (Becton Dickinson Life Sciences, Sparks, MD, USA). If the beta-lactamase result for *S. aureus* or NAS isolates contradicted the *bla*Z result obtained by PathoProof™ PCR from the sample, the isolates were screened for the *bla*Z gene by conventional PCR. The presence of the *mecA* gene was analysed with PCR for NAS isolates when the oxacillin MIC was ≥ 1 mg/L and for *S. aureus* when the cefoxitin MIC was ≥ 8 mg/L. If *mecA* was not detected, the isolate was further tested for the *mecC* gene.

Genomic DNA was isolated using InstaGene™ Matrix (Bio-Rad Laboratories, Hercules, CA, USA), and 1 µL of the supernatant was used as a PCR template. PCR amplification of *bla*Z with the expected product size of 518 bp was performed using previously described primers [15]. The PCR reaction mixture (25 µL) contained 1 µM of primers blaZ1 and blaZ2, 200 µM of deoxynucleoside triphosphate (Thermo Fisher Scientific, Vilnius, Lithuania), 2.5 µL of 10x thermophilic buffer and 0.5U of DNA polymerase (DreamTaq Polymerase, Thermo Fisher Scientific, Vilnius, Lithuania). The PCR product for *mecA* with the expected product size of 533 bp was amplified using previously described primers [16], the PCR reaction mixture containing 0.3 µM of forward and reverse primers. The presence of the *mecC* gene was analysed with the protocol of the European Union Reference Laboratory for Antimicrobial Resistance [17].

PCR amplification was performed in a Bio-Rad C1000 Touch Thermal Cycler (Bio-Rad Laboratories, Singapore) with the following cycling conditions: an initial denaturation for 5 min at 94 °C, 30 cycles of amplification (denaturation at 94 °C for 30 s, annealing at 55 °C (*blaZ* and *mecA*) or 59 °C (*mecC*) for 30 s, extension at 72 °C for 30 s (*blaZ* and *mecA*) or 1 min (*mecC*)), and a final extension at 72 °C for 10 min. The amplification products were analysed on 1.5% (*blaZ* and *mecA*) or 2% (*mecC*) (w/v) agarose gels (Sigma-Aldrich, St. Louis, MO, USA). *S. aureus* ATCC 29213, ATCC 43300 and MRSA_LGA251_ were used as positive controls for *bla*Z, *mecA* and *mecC*, respectively, and water as a negative control.

### Whole-genome sequencing and sequence analysis

Species identification of *S. borealis* was achieved through whole-genome sequencing by calculating the average nucleotide identity (ANI) with the OrthoANIu algorithm [18]. A threshold of 96% identity was used for species delineation [19].

*Klebsiella pneumoniae* and *E. coli* isolates were subjected to whole-genome sequencing to characterise the resistance mechanisms if the MIC value for colistin or 3rd generation cephalosporins (cefotaxime or ceftazidime) was above the ECOFF.

Bacterial genomic DNA was extracted using the QIAGEN DNeasy Blood & Tissue Kit (Qiagen GmbH, Hilden, Germany) according to the manufacturer’s instructions for Gram-positive bacteria. The purity of DNA was assessed with a DeNovix spectrophotometer (DeNovix Inc., Wilmington, DE, USA), accepting the extraction if the A260/280 ratio exceeded 1.8. A Qubit Fluorometer (Thermo Fisher Scientific, Malaysia) was used to measure the DNA concentration. Genomic libraries were prepared with the Illumina^®^ DNA Prep Kit (Illumina, San Diego, CA, USA) and paired-end sequencing (250 bp read length) was conducted on a MiSeq platform (Illumina, San Diego, CA, USA).

*De novo* assembly and quality control of the raw sequences were performed using the INNUca 4.2.2 pipeline [20]. In brief, the pipeline checked read quality using FastQC 0.11.5 [21] and trimmed the low quality reads using Trimmomatic 0.38 [22]. Assembly was performed with SPAdes 3.14 [23] and assembly correction with Pilon 1.23 [24].

The antibiotic resistance determinants were searched from assembled genomes using default settings in ResFinder 4.4.1 [25, 26], accessed on 27.11.2023 (*K. pneumoniae* isolates), and ResFinder 4.4.2 [25, 26], accessed on 31.1.2024 (*E. coli* isolate).

### Mapping the geographical locations of specimens

To create a map of the geographical locations of the milk specimens, the name of the isolate detected, and the name of the municipality were exported from the laboratory information system using Qlik Sense (Nov. 2023 Patch 9, QlikTech International AB). Municipality names were converted to coordinates using GPS Visualizer (https://www.gpsvisualizer.com/geocoder/). Municipality coordinates were weighted by the number of isolates, coloured by bacterial species or group and visualized with QGIS (v3.38.1, Free Software Foundation, Inc.).

## Results

Sampling packages were sent to 600 dairy farms, and a total of 1,786 milk samples were received from 238 (39.7%) farms (average 7.5 specimens/farm, range 1–20 specimens per farm). No pathogen was detected in 230/1,786 specimens, and three or more microbial findings were detected in 111/1,786 specimens. For milk samples with a microbe detected, on average, 1.4 microbial findings per specimen were recorded with PCR.

After removing ineligible specimens, a total of 863 milk samples from 801 individual cows and 220 different farms were cultured. The average age of the cows from which samples were cultured was 5.4 years, with a range of 1.8 to 15.4 years. Age information was not available for 28 cows (3.5%).

**Fig. 1:**
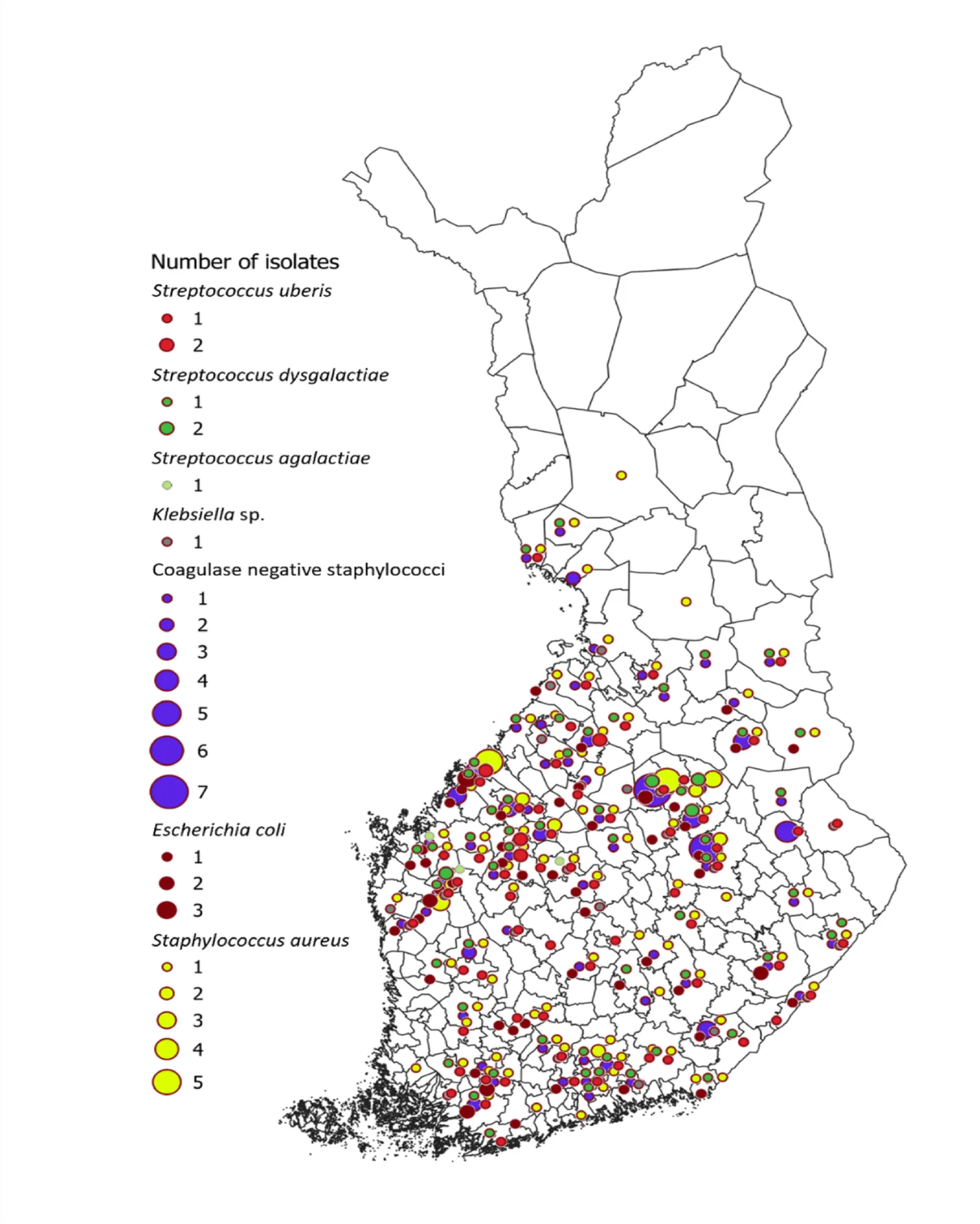
Geographical distribution of different bacterial isolates from clinical mastitis milk specimens in Finland, 2022–2023

After selecting only one isolate per bacterial species per farm, susceptibility testing was conducted for 502 bacterial isolates. The number of isolates per bacterial species is presented in Table 1. While the target number of *Streptococcus* sp. and *E. coli* isolates was reached, the number of *Staphylococcus aureus* and NAS isolates fell short of the goal. The geographical origin of the investigated specimens is illustrated in Fig. 1. Specimens mainly originated from those areas in Finland where the dairy industry is concentrated.

**Table 1:** The bacterial species and the number of isolates investigated compared with the number targeted

Species	*n* obtained	% of total	*n* targeted	% of targeted
*Staphylococcus aureus*	126	25	246	51
Non-aureus staphylococci (NAS), total	124	25	323*	38
*Staphylococcus epidermidis*	50	10	*	-
*Staphylococcus simulans*	23	5	*	-
*Staphylococcus haemolyticus*	18	4	*	-
*Staphylococcus chromogenes*	15	3	*	-
*Staphylococcus xylosus*	6	1	*	-
*Staphylococcus cohnii*	3	1	*	-
*Staphylococcus borealis*	2	0	*	-
*Staphylococcus hominis*	2	0	*	-
*Staphylococcus hyicus*	2	0	*	-
*Staphylococcus capitis*	1	0	*	-
*Staphylococcus gallinarum*	1	0	*	-
*Staphylococcus pasteuri* ^**^	1	0	*	-
*Streptococcus uberis*	79	16	73	108
*Streptococcus dysgalactiae*	74	15	73	101
*Escherichia coli*	70	14	73	95
*Klebsiella pneumoniae*	20	4	All	
*Streptococcus agalactiae*	6	1	All	
*Klebsiella oxytoca*	3	1	All	

*The target was to obtain a total of 323 NAS isolates

**MALDI score 1.92 [49]

Susceptibility results for *S. aureus*, NAS, *S. uberis*, *S. dysgalactiae*, *E. coli* and *Klebsiella* spp. are presented in Tables 2, 3, 4, 5, 6 and 7, respectively.

**Table 2:**
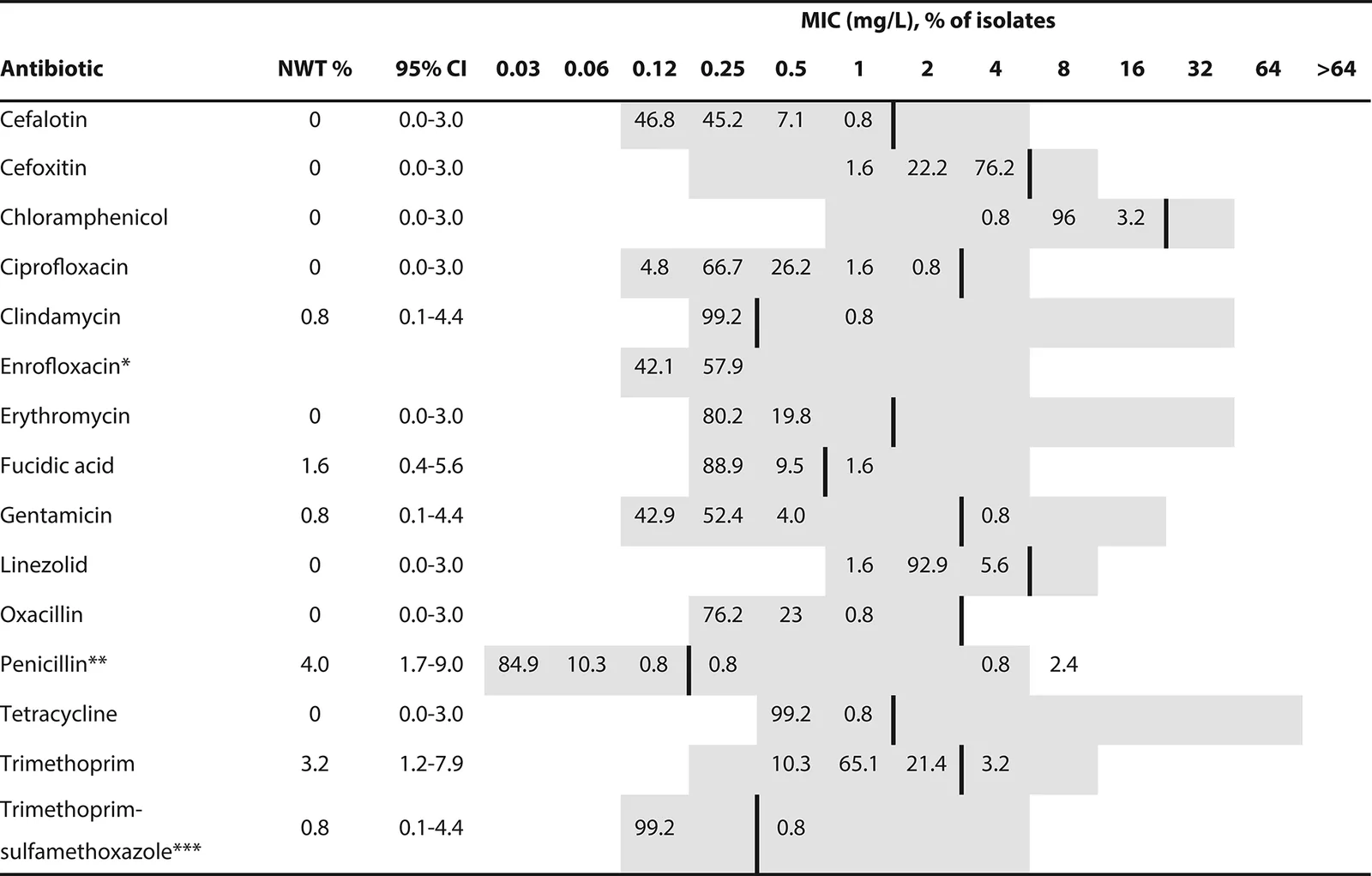
Susceptibility and MIC results for *Staphylococcus aureus* isolated from mastitis milk samples in Finland

The number of isolates tested was 126. A bold vertical line indicates a (t)ECOFF value. Grey shading indicates the tested interval.

MIC: minimum inhibitory concentration; NWT: non-wild type, isolates with a decrease in susceptibility; CI: confidence interval for NWT proportion

*No (t)ECOFF limits available

**The NWT proportion is based on beta-lactamase production

***Tentative ECOFF

Nearly all *S. aureus* isolates were classified as wild type (WT) to all tested antibiotics (Table 2). Notably, no methicillin-resistant *Staphylococcus aureus* (MRSA) isolates were discovered, as all isolates were classified as WT to cefoxitin (MIC range ≤ 0.25–4 mg/L) and oxacillin (MIC range ≤ 0.25–1 mg/L). Only 4% (95% CI: 1.7-9.0) of *S. aureus* isolates produced beta-lactamase, indicating resistance to penicillin. Eight original milk samples were positive for *bla*Z and *S. aureus*, which was the only staphylococcal species in these samples detected in the PathoProof™ test. However, *S. aureus* strains isolated from these samples neither produced beta-lactamase nor harboured the *bla*Z gene in conventional PCR.

Altogether, nine NAS isolates were classified as NWT to oxacillin: four of these were *S. epidermidis*, three *S. cohnii*, one *S. haemolyticus* and one *S. pasteuri* (Table 3). All four *S. epidermidis* isolates and the *S. haemolyticus* isolate were positive for *mecA*. None of the *mecA*-negative NAS harboured *mecC*. NAS isolates were mostly classified as WT to other antibiotics.

**Table 3:**
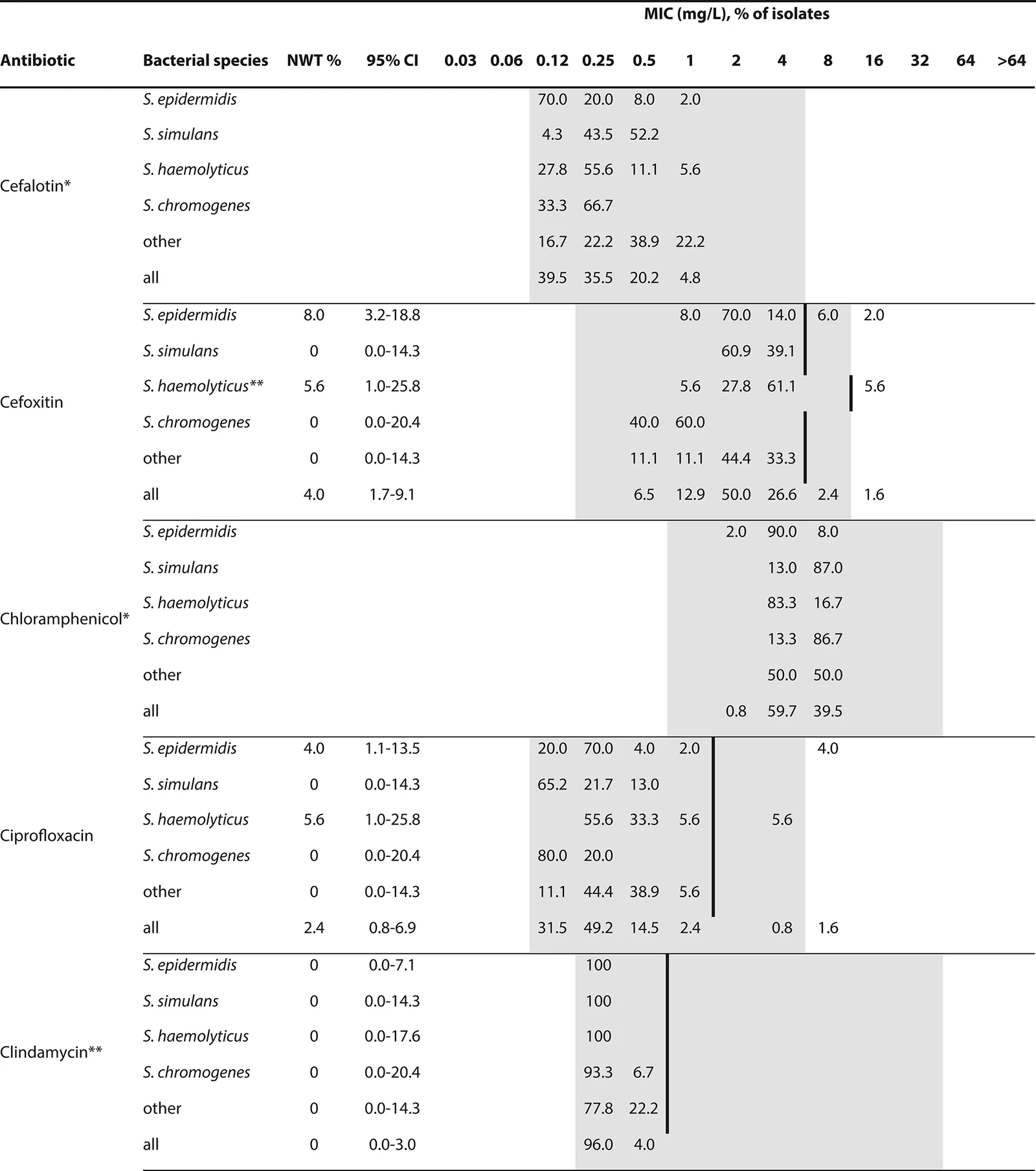

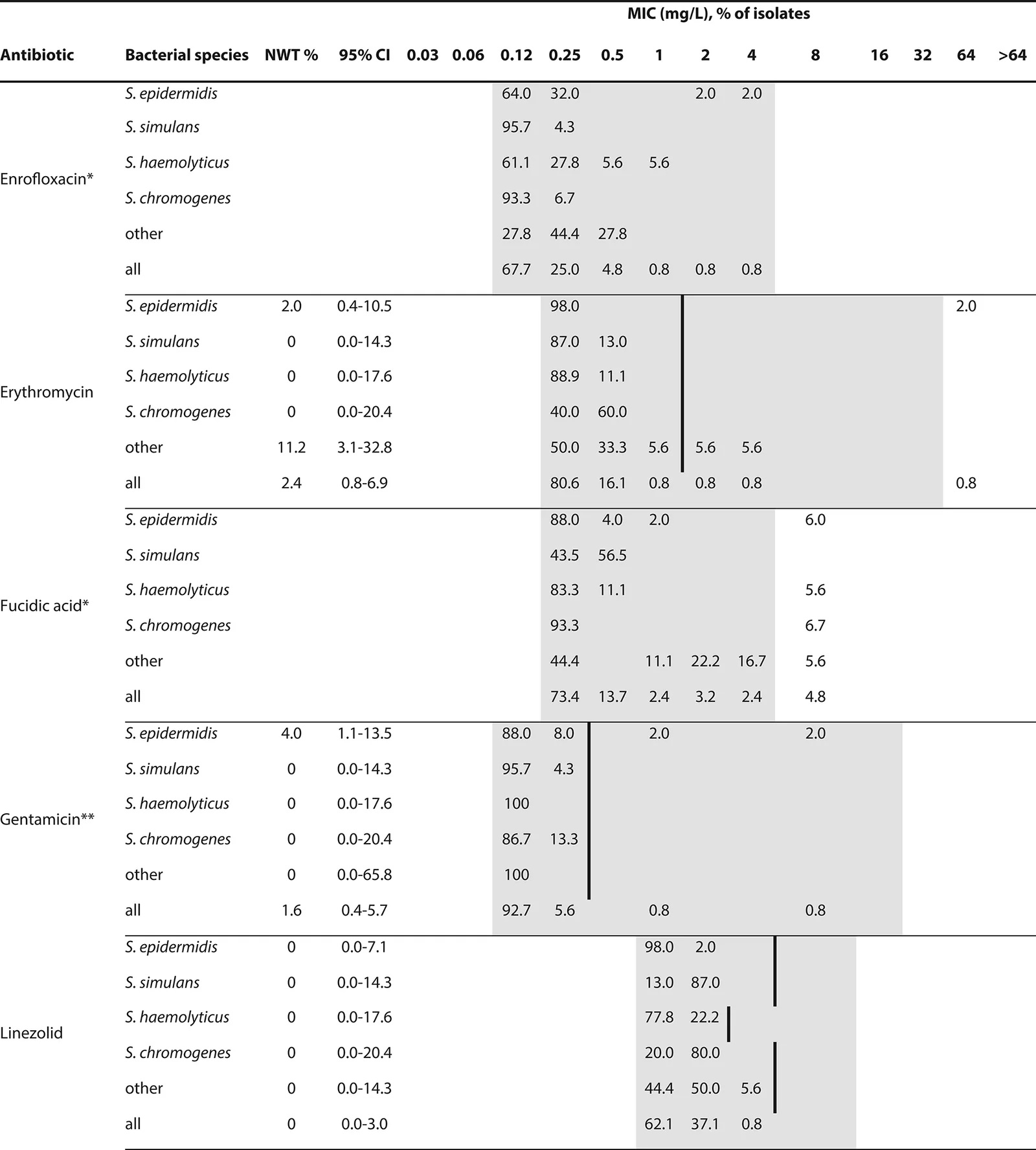

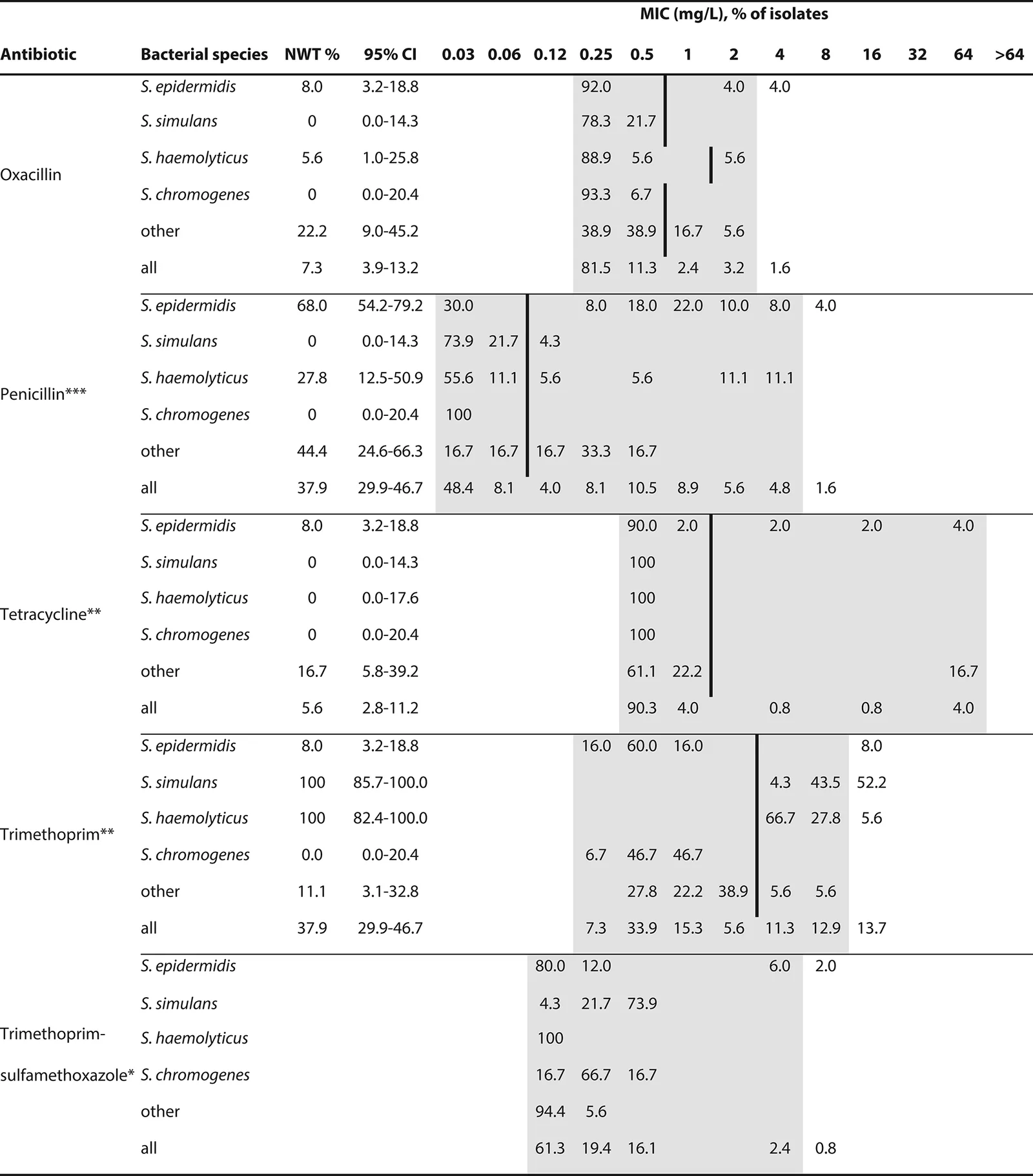
Susceptibility and MIC results for non-aureus staphylococci isolated from mastitis milk samples in Finland

The number of isolates tested: *S. epidermidis n*= 50, *S. simulans n*= 23, *S. haemolyticus n*= 18, *S. chromogenes n*= 15, other *n*= 18, all *n*= 124. A bold vertical line indicates a (t)ECOFF value for the four most common bacterial species. For other staphylococcal species, and if a species-specific (t)ECOFF was not available, a (t)ECOFF designated for *S. epidermidis *was used. The grey area indicates the tested interval.

MIC: minimum inhibitory concentration; NWT: non-wild type, isolates with a decrease in susceptibility; CI: confidence interval for NWT proportion

*No (t)ECOFF limits available

**Tentative ECOFF

***The NWT proportions are based on beta-lactamase production

Most NAS isolates did not produce beta-lactamase, but the proportion varied considerably according to the bacterial species. Sixty-eight percent (95% CI: 54.2–79.2) of *S. epidermidis* isolates produced beta-lactamase, and all of them were classified as NWT to penicillin in broth microdilution. One *S. epidermidis* isolate that was classified as NWT to penicillin and oxacillin was negative in the beta-lactamase test. Of all *S. haemolyticus* isolates, 28% (95% CI: 12.5–50.9) were positive in beta-lactamase testing, while according to MIC testing, 33% (95% CI: 16.3–56.3) were categorised as NWT. Beta-lactamase-producing staphylococci were also detected among *S. xylosus* (*n* = 3), *S. cohnii* (*n* = 3), *S. hominis* (*n* = 1) and *S. gallinarum* (*n* = 1). Discrepancies between the beta-lactamase test result and classification as NWT based on the *S. epidermidis* ECOFF (> 0.06 mg/L) were related to isolates with a penicillin MIC slightly above the ECOFF (0.12–0.25 mg/L).

Ten NAS isolates were tested for the *blaZ* gene by conventional PCR due to a discrepancy between beta-lactamase production of the strain and the *blaZ* result obtained with the PathoProof™ test from the milk sample. Based on the PathoProof™ test, these milk samples were negative for *S. aureus*. Of these, eight NAS isolates obtained through culturing did not produce beta-lactamase and were negative for *bla*Z by conventional PCR, while the milk samples were positive for the *blaZ* gene with the PathoProof™ test. On one occasion, a positive *blaZ* result obtained with the PathoProof™ test was confirmed by conventional PCR of the NAS (*S. epidermidis*) isolate, although the strain was negative in the beta-lactamase test. Additionally, one *S. gallinarum* isolate was positive in the beta-lactamase test, contradicting the negative *blaZ* results obtained with both the PathoProof™ test and conventional PCR.

A sizeable proportion of the *S. uberis* isolates were classified as NWT to penicillin (42%, 95% CI: 31.5–52.8) and tetracycline (38%, 95% CI: 29.2–50.3) (Table 4). While no cut-off values are available for oxacillin or cefalotin, MICs were notably quite high. A cut-off value is lacking for penicillin and *S. dysgalactiae*, but MIC values of all the isolates were low (0.03 mg/L). Among *S. dysgalactiae*, only tetracycline displayed a wide MIC distribution (Table 5). Only six *S. agalactiae* isolates were tested. All displayed WT MIC values for all tested antibiotics except tetracycline. A tetracycline NWT phenotype was detected in three of the six *S. agalactiae* isolates.

**Table 4:**
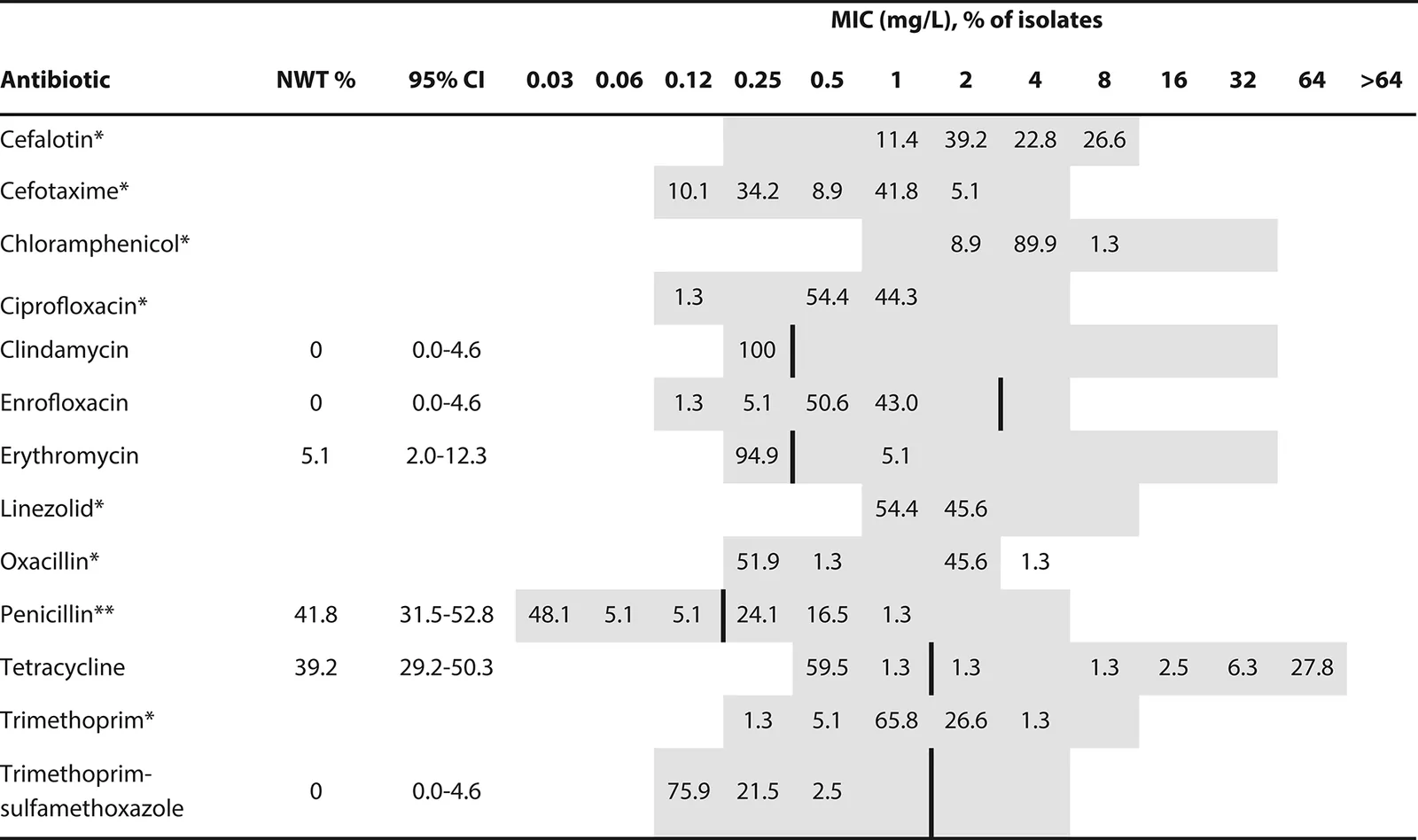
Susceptibility and MIC results for *Streptococcus uberis* isolated from mastitis milk samples in Finland

The number of isolates tested was 79. A bold vertical line indicates a (t)ECOFF value. Grey shading indicates the tested interval.

MIC: minimum inhibitory concentration; NWT: non-wild type, isolates with a decrease in susceptibility; CI: confidence interval for NWT proportion

*No (t)ECOFF limits available

**Tentative ECOFF

**Table 5:**
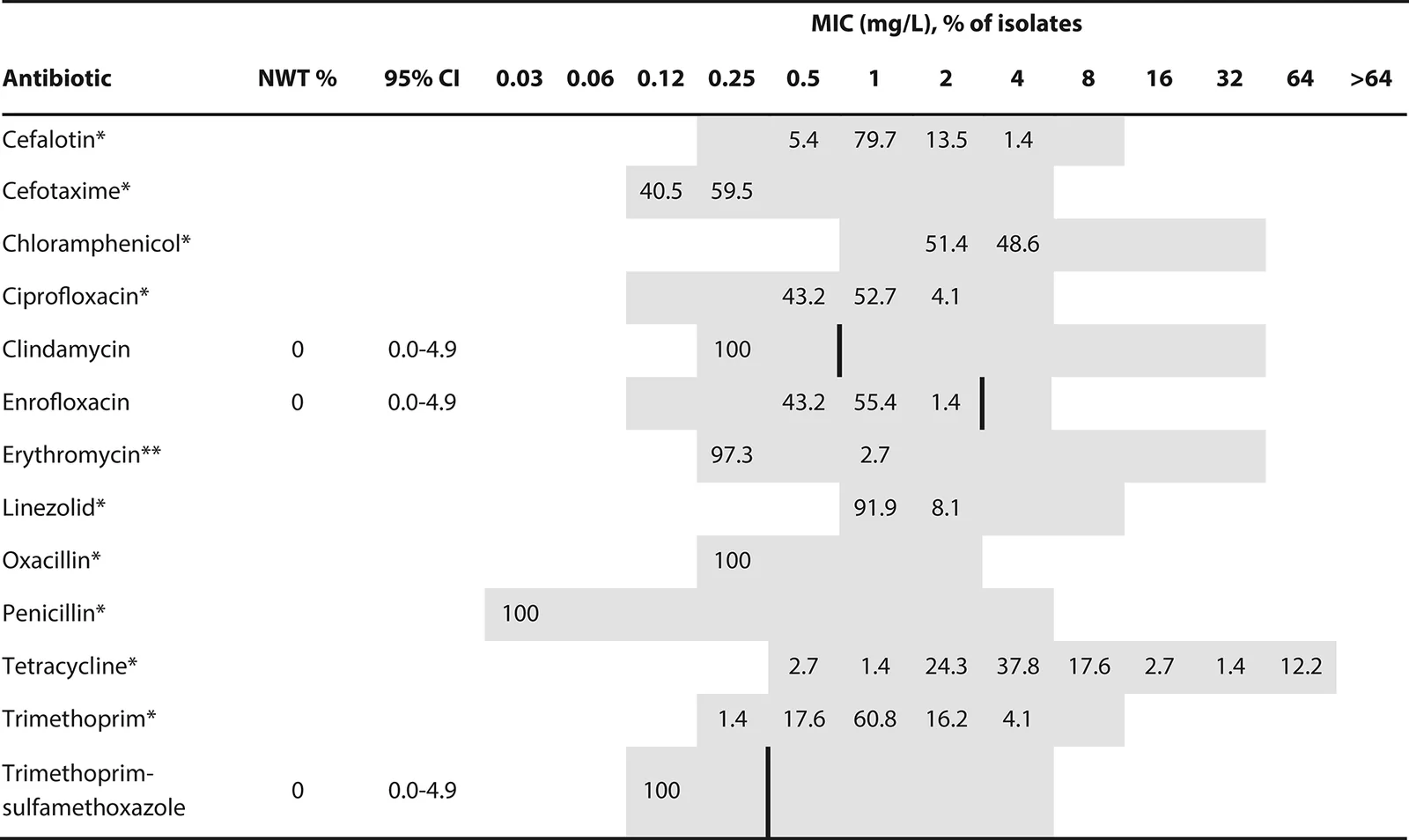
Susceptibility and MIC results for *Streptococcus dysgalactiae* isolated from mastitis milk samples in Finland

The number of isolates tested was 74. A bold vertical line indicates an ECOFF value. Grey shading indicates the tested interval.

MIC: minimum inhibitory concentration; NWT: non-wild type, isolates with a decrease in susceptibility; CI: confidence interval for NWT proportion

*No (t)ECOFF limits available

**Concentrations tested do not allow the calculation of NWT proportion with the current ECOFF (> 0.125 mg/L)

*E. coli* and *Klebsiella* spp. displayed similar proportions of NWT isolates to trimethoprim (7% vs. 9%, respectively) and trimethoprim-sulfamethoxazole (7% vs. 9%), as well as to tetracycline (14% vs. 13%) (Tables 6 and 7). *E. coli* isolates were otherwise generally classified as WT, apart from ampicillin. One *E. coli* isolate with elevated MIC values for cefotaxime (2 mg/L) and ceftazidime (4 mg/L) had an AmpC promoter mutation C42T. The assembled genomes of the two *Klebsiella pneumoniae* isolates with colistin MICs of 4 mg/L and > 8 mg/L were analysed through ResFinder to search for resistance determinants. The analysis found *bla*_SHV_, *fosA*, *oqx*A and *oqx*B genes with 94.8–99.9% identity and 100% coverage from both isolates, and *tetA* (100% identity and coverage) from one isolate, together with several chromosomal point mutations in *acrR*, *ompK36* and *ompK37* (Table 8). No *mcr* genes were found.

**Table 6:**
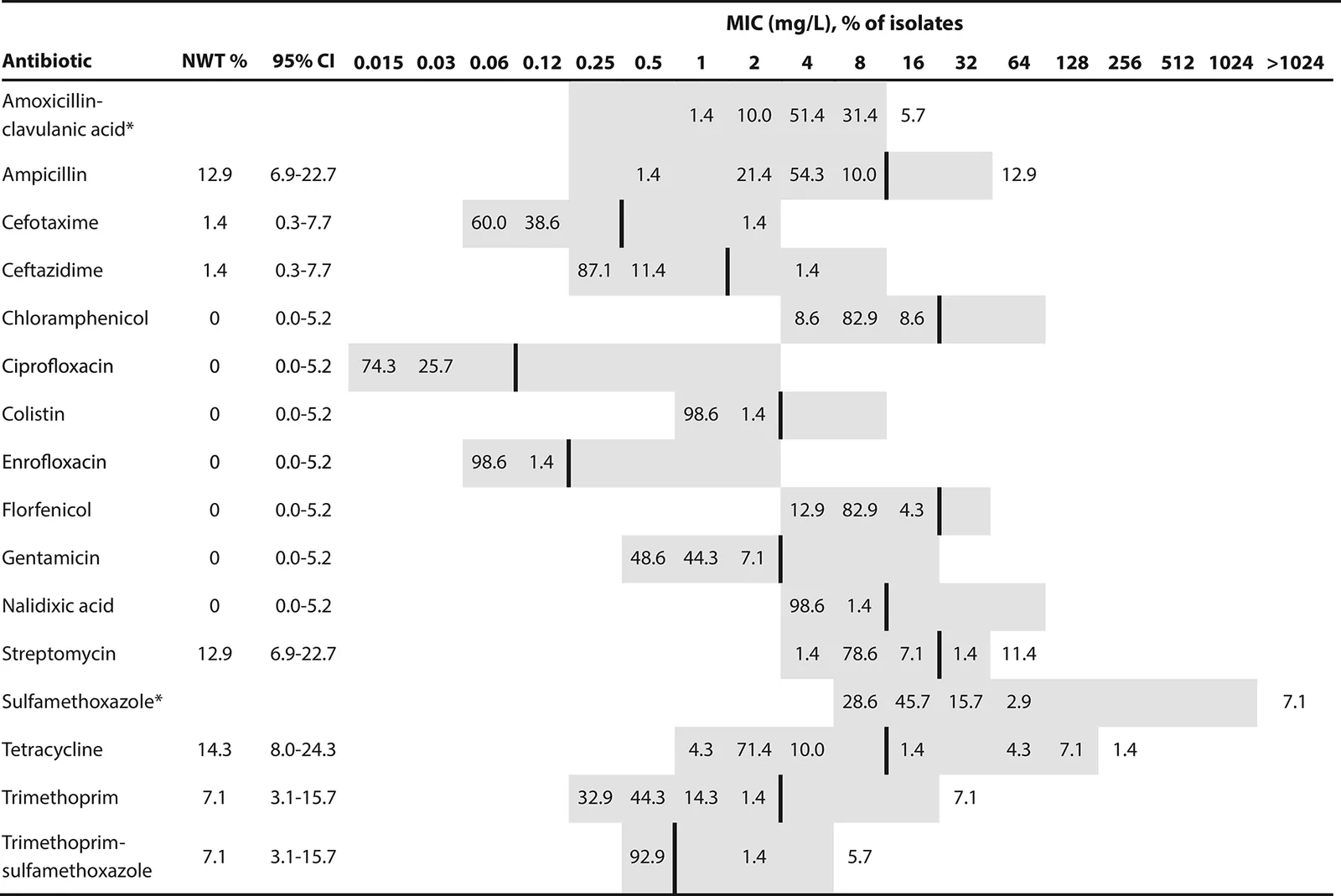
Susceptibility and MIC results for *Escherichia coli* isolated from mastitis milk samples in Finland

The number of isolates tested was 70. A bold vertical line indicates an ECOFF value. Grey shading indicates the tested interval.

MIC: minimum inhibitory concentration; NWT: non-wild type, isolates with a decrease in susceptibility; CI: confidence interval for NWT proportion

*No (t)ECOFF limits available

**Table 7:**
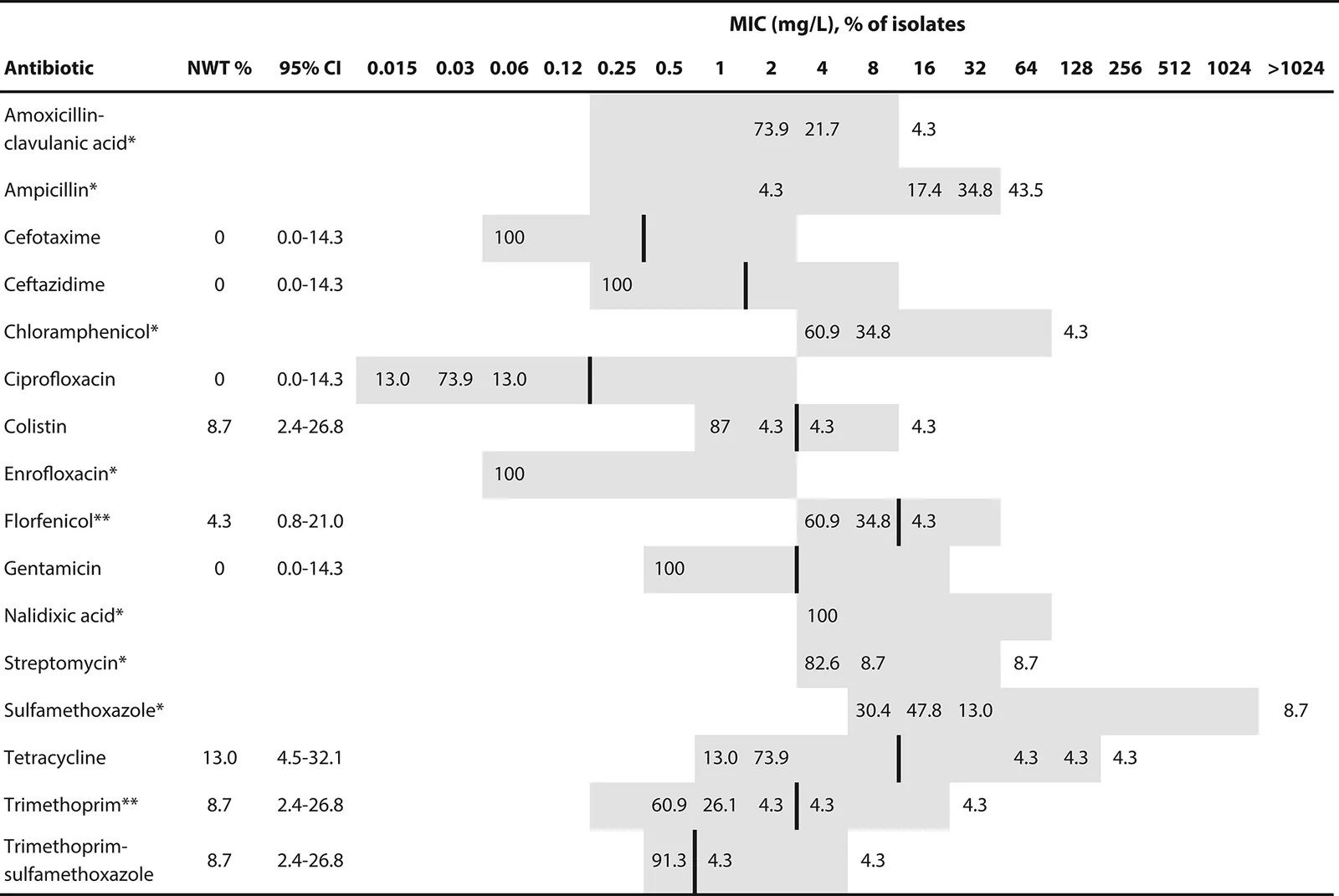
Susceptibility and MIC results for *Klebsiella *sp. isolated from mastitis milk samples in Finland

The number of isolates tested was 23. A bold vertical line indicates a (t)ECOFF value for *K. pneumoniae*. Grey shading indicates tested interval.

MIC: minimum inhibitory concentration; NWT: non-wild type, isolates with a decrease in susceptibility; CI: confidence interval for NWT proportion

*No (t)ECOFF limits available

**Tentative ECOFF

**Table 8:** Acquired resistance genes and point mutations in *Klebsiella pneumoniae* isolates NWT* to colistin

Strain ID	NWT phenotype	Resistance gene (identity, coverage)	Known chromosomal point mutations
FIAR-2636	Colistin, Tetracycline	*bla*_SHV−89/85/79/56/40_ (99.88%, 100%), *fosA5* (94.76%, 99.76%), *oqxA* (99.74%, 100%), *oqxB* (98.92%, 100%), *tetA* (100%, 100%)	*acrR* (p. P161R, p. G164A, p. F172S, p. R173G, p. L195V, p. F197I, p. K201M), *ompK36* (p. N49S, p. L59V, p. T184P), *ompK37* (p. I70M, p. I128M)
FIAR-2653	Colistin	*bla*_SHV−187_ (99.54%, 100%), *fosA6* (99.52%, 100%), *oqxA* (99.40%, 100%), *oqxB* (99.49%, 100%)	*acrR* (p. P161R, p. G164A, p. F172S, p. R173G, p. L195V, p. F197I, p. K201M), *ompK36* (p. N49S, p. L59V, p. T184P), *ompK37* (p. I70M, p. I128M, p. N230G)

*NWT phenotype based on MIC values

## Discussion

This study aimed to determine the antibiotic susceptibility of the most common mastitis pathogens in dairy cows in Finland. Epidemiological cut-off values, which are used to identify non-wild type phenotypes from wild type organisms, were chosen to this study since they allow better prediction of acquired resistance mechanisms. It should be noted, however, that ECOFFs do not define clinical resistance. The lack of epidemiological cut-off values, and differences in susceptibility testing methodologies as well as in the interpretation criteria used in other studies, made explicit reporting challenging. Consequently, comparison of our results to the previous studies is descriptive. However, the overall proportion of NWT isolates for bacteria–antibiotic combinations for which epidemiological cut-offs were available was very low. In cases where no ECOFF values existed, MIC values were mainly low.

Penicillin is the primarily recommended [27] and most commonly used antibiotic [28] in Finland. Only 4% (95% CI: 1.7-9.0) of *S. aureus* isolates in our study produced beta-lactamase, exhibiting phenotypic penicillin resistance, and an overall decrease from previous studies in Finland is noticeable. Roughly 25% of *S. aureus* isolates from clinical mastitis cases produced beta-lactamase in 2005 [29] and 2012 [30], while the proportion of resistant isolates from subclinical mastitis specimens was 52% in 2001 [31]. The decreasing trend has continued, as a more recent study from Finland detected beta-lactamase producing *S. aureus* in 12% of the isolates from milk samples also sent to and tested in the mastitis laboratory of Valio Ltd in 2017 [32]. However, the results are not fully comparable between studies, because the study designs have been different. For example, isolate selection, sample collection and geographical representativeness have differed between studies. The proportion of isolates resistant to penicillin in our study is now more similar to that in Sweden, where penicillin resistance was reported to be around two to three percent in 2019–2023 [33]. A Danish study in 2019 determined the penicillin resistance of *S. aureus* to be nearly 18% among 63 isolates [3], which is far above what was revealed in our study, while the proportion of resistant isolates in Norway was lower, being only 2.5% [34]. A recent meta-analysis found the penicillin resistance of *S. aureus* to be 45% [9]. The same study revealed that penicillin resistance was highest in Africa and Asia (66–70%), while studies from Europe and North America (40–45%) demonstrated moderate resistance. It is noteworthy that according to the global meta-analysis [9], resistance increased from 43% prior to 1999 to 65% in 2014–2020, while penicillin resistance in *S. aureus* appears to have displayed an opposite trend in Finland.

*S. epidermidis* was by far the most prevalent NAS species, comprising 40% of the isolates, which is similar to Norway [34], where 43% (531/1,236) of NAS isolates were *S. epidermidis*. Conversely, *S. epidermidis* only comprised 18% (9/49) of the NAS isolates in Denmark, with *S. simulans* (27%, 13/49) and *S. chromogenes* (22%, 11/49) being more common [3]. All of these are, however, among the most frequently isolated NAS in dairy mastitis [35]. In this study, we also detected two isolates of *S. borealis*, which is a newly identified mastitis pathogen [36]. *S. borealis* was identified with MALDI-TOF, and the species was further confirmed with WGS. Resistance to penicillin based on beta-lactamase production among NAS was 38%, but it varied considerably between 0 and 68%, depending on the four most common species. This level is the same as in FINRES-Vet national antimicrobial resistance monitoring in 2012 [30], but slightly higher than the results for non-aureus staphylococci (NAS) in Norway [34], where 34% of isolates from dairy mastitis cases were resistant to penicillin in the cloverleaf assay for beta-lactamase production. However, only 22% of NAS isolates in Denmark were penicillin resistant by broth microdilution [3], a far lower proportion than revealed in our study. In a recent study from Finland comparing the susceptibility of *S. aureus* and NAS from mastitis specimens, discordant results were obtained for the presence of the *bla*Z gene in the milk sample based on PathoProof™ PCR, inhibition-zone-based penicillin susceptibility and a nitrocefin beta-lactamase production test on the isolates [32]. In that study, 79.6% of *S. epidermidis* isolates carried the *bla*Z gene, while 70.2% were resistant according to penicillin disk diffusion and only 63.3% produced beta-lactamase in the nitrocefin test. Our result (68% penicillin resistance among *S. epidermidis* isolates based on beta-lactamase production) fits very well with the disk-diffusion results. Despite differences in the methods and interpretative criteria used, penicillin resistance has been very common among *S. epidermidis* in Finland, with the occurrence being between 70% and 80% [32, 37]. For other NAS species, the proportions of isolates producing beta-lactamase differed among both *S. chromogenes* and *S. simulans*, as our results demonstrated no resistance compared to 19% and 5%, respectively, reported previously [32].

It should be noted that PCR results (the presence of *blaZ* gene) obtained with the PathoProof™ test might not correctly indicate penicillin resistance among *S. aureus* and NAS strains isolated from the milk specimens. In our study, 16 samples yielded a positive result for the *blaZ* gene in the PathoProof™ PCR test, although *S. aureus* (*n* = 8) or NAS (*n* = 8) obtained from these samples were negative for the *blaZ* gene in conventional PCR and did not produce beta-lactamase. Possible explanations might be that the milk specimens contained other staphylococci in very low quantities or remnants of their genes, as the PathoProof™ PCR is not able to determine in which bacterial species the *blaZ* gene is located. Furthermore, contradictory results for nitrocefin beta-lactamase production and the presence of the *blaZ* gene in the cultured isolate are possible but were less frequent in our study.

In our study, methicillin resistance among NAS was uncommon and similar to findings previously reported from Finland [32, 38]. Moreover, MRSA was not detected in our study, and it has only been reported in a few clinical mastitis cases in Finland [32, 38].

The most concerning finding was the proportion of *S. uberis* isolates that were classified as NWT to penicillin (42% based on a tentative ECOFF of > 0.125 mg/L, 95% CI; 29.9–46.7). The NWT prevalence of this pathogen has increased during the past ten years, reported as only 4% in 2012 [30]. Other studies have demonstrated wide variation in the susceptibility of *S. uberis* isolates from mastitis specimens. An increase in the proportion of *S. uberis* isolates classified as NWT to penicillin has also been noted in Denmark, as NWT proportions using a tentative ECOFF of > 0.125 mg/L were 18% in 2016 and over 50% in 2018–2021 [3, 39]. On the contrary, the proportion of NWT *S. uberis* isolates was only 6.4% in 2020–2021 in Norway, a considerably lower proportion than in our study [39]. Furthermore, in Sweden, among *S. uberis* isolates collected between 2013 and 2020, the proportion of isolates with an MIC of > 0.125 mg/L was small (< 1%) [40]. Our results are more in line with the UK VARSS report for bulk tank milk samples, in which 42% of *S. uberis* isolates were classified as NWT to penicillin [41]. It is noteworthy that in the UK VARSS report, among isolates from clinical mastitis (*n* = 26), none were penicillin resistant, but in this case, different methodology and interpretative criteria were used. Other studies have also shown high proportions of penicillin NWT *S. uberis* isolates. In a recent study in the Czech Republic, 75% of isolates would be categorised as NWT when using a tentative ECOFF of > 0.125 mg/L [42]. The penicillin MICs of the *S. uberis* isolates found in this study were lower than the benzylpenicillin concentrations detected in milk from healthy animals [43]. However, whether the elevated penicillin MIC values of *S. uberis* impact the efficiency of penicillin in the treatment of mastitis remains unclear due to lack of intramammary pharmacokinetic-pharmacodynamic and clinical outcome data. The resistance mechanism for the elevated penicillin MICs has been suggested to be linked with mutations in penicillin-binding proteins [40, 44, 45]. In our isolates, MIC values were as high as 1 mg/L, and further investigations are needed to determine the underlying mechanisms.

No penicillin NWT phenotypes were detected among *S. dysgalactiae* isolates in our study. This is in line with data from both Denmark and Norway [39]. However, the proportion of tetracycline NWT isolates was notable in our data, being nearly 34% when using a limit of > 4 mg/L. With the previously proposed limit of 8 mg/L [39], the NWT proportion in our study would have decreased to 16.3%. Furthermore, it was reported that 0% and 22% of the isolates exhibited an NWT phenotype to oxytetracycline using the 8 mg/L limit for isolates from Norway and Denmark, respectively.

In general, Enterobacteriaceae investigated in this study were mostly classified as WT to the tested antibiotics when ECOFFs were available. While supportive treatment is the first-line therapy for *E. coli* mastitis in national recommendations, all isolates were classified as WT to enrofloxacin, the alternative, secondary treatment [27]. The proportions of tetracycline NWT *E. coli* and *Klebsiella* spp. isolates were similar as reported in Denmark [3] for *E. coli*. However, no tetracycline NWT isolates (MICs > 8 mg/L) was detected in *Klebsiella* sp. isolates in the same study [3].

In this study, two colistin NWT *Klebsiella pneumoniae* isolates were further analysed with WGS to assess whether acquired resistance genes, especially mobile colistin resistance (*mcr)* genes, could explain the observed MIC values. ResFinder analysis did not reveal *mcr* genes. Colistin resistance in *K. pneumoniae* can, however, be mediated by more commonly observed mechanisms, such as alterations in chromosomally located genes (*mgrB*, *phoPQ*, *pmrAB* and *crrAB*), upregulated expression of efflux pumps and overproduction of capsular polysaccharides [46, 47], which cannot be analysed with ResFinder. Therefore, further studies would be needed to explore the genetic background of our isolates. Although colistin is banned in veterinary medicine in Finland, monitoring of colistin resistance and the underlying mechanisms is important in a One Health perspective, as it is considered one of the last-resort antibiotics in the treatment of infections caused by multidrug-resistant Gram-negative bacteria in humans.

One of the limitations of this study was that the desired number of isolates could not be obtained, especially for staphylococci, thus impacting the precision of the estimates. Although the target number of *S. aureus* isolates was not achieved, the number of isolates (*n* = 126) obtained enabled an assessment of the proportion of isolates resistant to penicillin with the desired 5% precision due to the lower prevalence of resistance than initially anticipated. For NAS, the proportion of beta-lactamase producing isolates varied considerably between species. For *S. uberis*, the proportion of penicillin NWT isolates was higher than expected, and the precision of our estimate was not therefore as good as we aimed to obtain. It is noteworthy that only a tentative ECOFF was available for penicillin and *S. uberis*, therefore, there may be more uncertainty regarding its accuracy. Regardless, the study provided new, important information about the penicillin susceptibility of *S. uberis* in Finland. Clinical relevance of our results is unknown since the use of epidemiological cut-off values does not predict clinical efficacy. However, the purpose of this survey was to evaluate developments in MIC distributions, and to identify potentially new emerging phenotypes. Of all the invited farms, 40% decided to participate and sent milk samples for analysis. This may have caused some selection bias. The farms were selected from all eligible farms through stratified random sampling based on geographical location. The participating farms represented well the overall geographical distribution of dairy farms in Finland. It is, however, possible that farms with more prudent antibiotic use or, on the other hand, farms with low cure rates, potentially due to AMR, were more likely to participate. This makes it hard to anticipate the potential direction of this bias. Data on the clinical severity of the cases was not collected. However, selecting samples for culture with the PCR preselection criteria (pathogen detected with ++ or +++ according to PathoProof™ software, and sample positive for at most two bacterial species), we aimed to confirm that the isolates included were clinically relevant as described earlier [48].

PCR methods, such as the one used to screen potential specimens for this study, have largely replaced the traditional culture and susceptibility testing of milk specimens in Finland [8]. While these methods are convenient, efficient, timesaving and cost effective, the downside is their inability to screen for the vast majority of antibiotic resistance. It is therefore pertinent to examine the antibiotic susceptibility of mastitis pathogens at least intermittently to maintain an understanding of the threat of resistance in these isolates and to guide policy decisions and treatment recommendations.

## Conclusions

Isolates from dairy cow mastitis classified as NWT to different antibiotics are generally rare, whereas beta-lactamase production was commonly detected among NAS isolates. In addition, the proportion of *Streptococcus uberis* isolates classified as NWT to penicillin has increased over the past ten years, and continued surveillance is warranted.

## Supplementary Information

Below is the link to the electronic supplementary material.

Supplementary Material 1.

## Data Availability

The datasets used and/or analysed during the current study are available from the corresponding author upon reasonable request.

## Declarations

### Prior publication

The data have not been published previously.

## Abbreviations

AMR: Antimicrobial resistance
ANI: Average nucleotide identity
CFU: Colony forming unit
CI: Confidence interval
DCT: Dry-cow therapy
ESBL: Extended-spectrum beta-lactamase
IMI: Intramammary infections
MALDI-TOF: Matrix-assisted laser desorption ionization time-of-flight
MIC: Minimum inhibitory concentration
MRSA: Methicillin-resistant *Staphylococcus aureus*
NAS: Non-aureus staphylococci
NWT: Non-wild type
(t)ECOFF: (Tentative) epidemiological cut-off
PCR: Polymerase chain reaction
SCC: Somatic cell count
WGS: Whole genome sequencing
WT: Wild type
