# Supplementary material for: Antibiotic susceptibility of mastitis pathogens in Finnish dairy cows in 2022–2023: concerning changes in the penicillin susceptibility of Streptococcus uberis
Source: Acta Vet Scand. 2026 Aug 21;68:42. doi: 10.1186/s13028-026-00875-4 (PMC13613593; doi:10.1186/s13028-026-00875-4)
Supplement: Supplementary file 1 [file 13028_2026_875_MOESM1_ESM.docx]

**Additional file 1.** Antimicrobials tested for each bacterial species isolated from bovine mastitis specimens in Finland 2022-2023.

| **Antimicrobial** | *Escherichia coli* | *Klebsiella* spp. | *Staphylococcus* spp. | *Streptococcus* spp. |
| --- | --- | --- | --- | --- |
| Amoxicillin-clavulanic acid | x | x | - | - |
| Ampicillin | x | x | - | - |
| Ciprofloxacin | x | x | x | x |
| Cefoxitin | - | - | x | x |
| Cefotaxime | x | x | - | - |
| Ceftazidime | x | x | - | - |
| Cephalotin | - | - | x | x |
| Chloramphenicol | x | x | x | x |
| Clindamycin | - | - | x | x |
| Colistin | x | x | - | - |
| Enrofloxacin | x | x | x | x |
| Erythromycin | - | - | x | x |
| Florfenicol | x | x | - | - |
| Fusidic acid | - | - | x | - |
| Gentamicin | x | x | x | - |
| Linezolid | - | - | x | x |
| Nalidixic acid | x | x | - | - |
| Oxacillin | - | - | x | x |
| Penicillin | - | - | x | x |
| Streptomycin | x | x | - | - |
| Sulfamethoxazole | x | x | - | - |
| Tetracycline | x | x | x | x |
| Trimethoprim | x | x | x | x |
| Trimethoprim-sulfamethoxazole | x | x | x | x |

x: tested; -: not tested.
